# Missing IUD Despite Threads at the Cervix

**DOI:** 10.1155/2014/916143

**Published:** 2014-08-27

**Authors:** Andrew L. Atkinson, Jonathan D. Baum

**Affiliations:** Department of Obstetrics and Gynecology, Jersey Shore University Medical Center, 1945 Route 33, Neptune, NJ 07753, USA

## Abstract

Today, the intrauterine device (IUD) is by far the most popular form of long term reversible contraception in the world. Side effects from the IUD are minimal and complications are rare. Uterine perforation and migration of the IUD outside the uterine cavity are the most serious complications. Physician visualization and/or the patient feeling retrieval threads at the cervical os are confirmation that the IUD has not been expelled or migrated. We present a case of a perforated, intraperitoneal IUD with threads noted at the cervical os. Office removal was not possible using gentle traction on the threads. Multiple imaging and endoscopic modalities were used to try and locate the IUD including pelvic ultrasound, diagnostic hysteroscopy, cystoscopy, and pelvic magnetic resonance imaging (MRI). The studies gave conflicting results on location of the IUD. Ultimately, the missing IUD was removed via laparoscopy.

## 1. Introduction

The modern intrauterine device (IUD) was first described in 1909 by Dr. Richard Richter, a physician who saw the need for reliable, long term, reversible contraception [1]. Currently, there are an estimated 180 million IUD users worldwide making it the most popular form of reversible contraception [2, 3]. Perforation of the uterus with subsequent migration into the peritoneal cavity or retroperitoneum is an uncommon but serious complication [4].

Andersson et al. reported a perforation rate of 1.3 per 1000 IUDs placed [5]. Risk factors for perforation include clinician inexperience, fixed or retroverted uterus, placement during lactation, or the presence of a defect in the myometrium [6]. Typically, the misplaced IUD is signaled by shortening or disappearance of retrieval threads at the cervical os.

We present a case of a perforated, intraperitoneal IUD with threads noted at the cervical os.

## 2. Case Presentation

34-year-old Caucasian female para 3 underwent levonorgestrel-releasing intrauterine device (LNG-IUD) placement three months following her third cesarean delivery. She was breastfeeding, had no significant medical history, and had no contraindications to an IUD. Placement of the IUD was unremarkable and examination showed two threads at the cervix approximately 2 centimeters (cm) in length following her next menstrual cycle. Subsequent menstrual cycles were regular and lasted 4-5 days. Overall, her bleeding pattern was regular with less flow than her typical menses; however, she did report intermenstrual spotting for up to 7 days a month. IUD threads remained visible at subsequent gynecologic visits with no other reported issues. She presented to the office two years after IUD placement complaining of acute onset, dull, achy pelvic pain. She was also experiencing vaginal spotting for the past two weeks prior to presentation. Pelvic exam confirmed two threads still approximately 2 cm in length at the cervical os. Office removal was not possible using gentle traction on the threads. Pelvic ultrasound showed the right arm of the IUD was imbedded within the myometrium of the lower uterine segment 3 millimeters from the serosa. The body of the IUD and the left arm were within the endometrial cavity. While pelvic ultrasound suggested the IUD was partially imbedded but still within the uterus, there was concern that the IUD had perforated her cesarean scar.

Diagnostic hysteroscopy showed an empty endometrial cavity with threads coursing within the endocervix and then disappearing posteriorly into the right lower uterine segment. The IUD was not seen within the uterine cavity nor was it seen on cystoscopy. Rectal exam confirmed an intact rectovaginal septum. The patient was informed of the operative findings and our inability to locate the IUD. The concern was an intramural location of the IUD which would require either wedge resection or hysterectomy. We chose MRI without contrast to locate the IUD.

**Figure 1:**
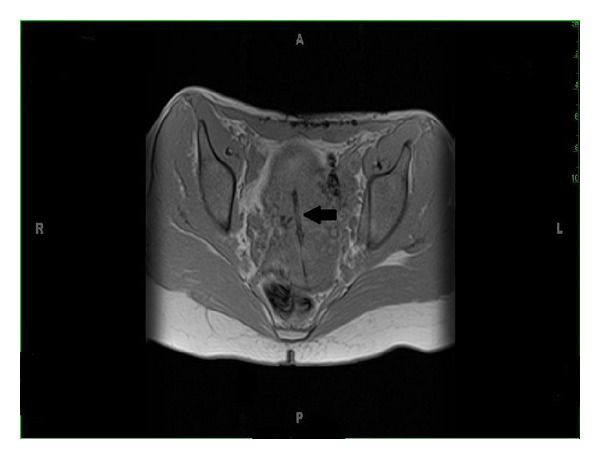
Arrow pointing to IUD within the uterus in a T1 weighted MRI, axial view.

Pelvic MRI showed that the body of the IUD was within the endometrial cavity with the right arm perforating the myometrium (Figure 1). The left arm was not seen suggesting that it had either collapsed or broken off.

**Figure 2:**
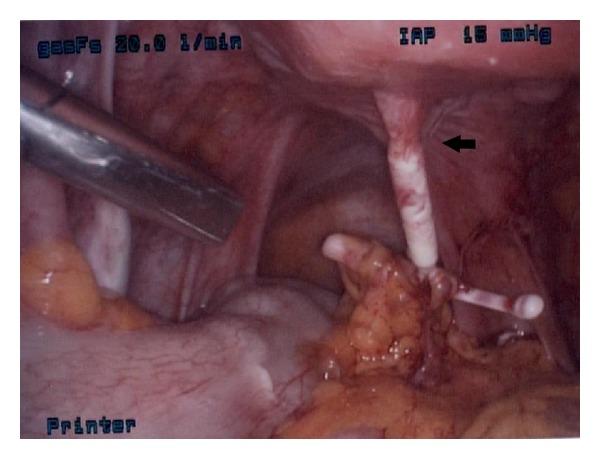
Arrow pointing to eye of IUD encased in fibrotic tissue. Arms of IUD covered in adhesions stemming from mesenteric adipose.

**Figure 3:**
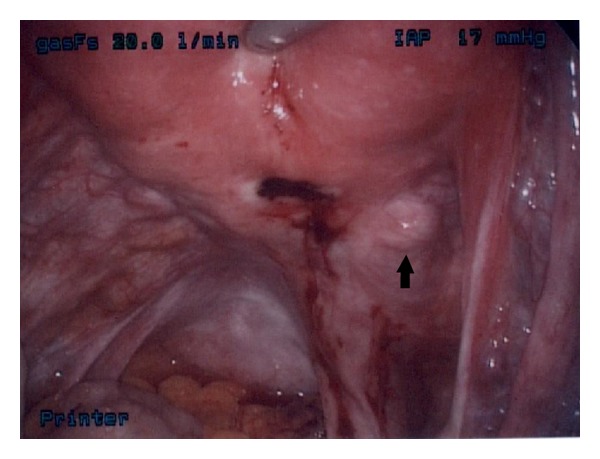
Arrow pointing to right uterine artery approximately 1 cm from the site of perforation.

Diagnostic laparoscopy was performed and the IUD was found posterior to the uterus in the peritoneal cavity encased in filmy and vascular adhesions to the mesenteric adipose (Figure 2). The IUD had perforated through the lower uterine segment about 1 cm from the right uterine artery (Figure 3). The eye of the IUD was trapped within serosal fibrosis. Following adhesiolysis and dissection, the IUD was removed with intact threads.

## 3. Discussion

A misplaced IUD is usually signaled by shortening or disappearance of its retrieval threads [7]. The presented case is unusual since IUD perforation was diagnosed despite threads remaining at a consistent length at the cervical os. The patients bleeding pattern and two years of effective contraception following IUD insertion were consistent with intrauterine placement. While sudden onset or heavy bleeding may indicate IUD expulsion or perforation, intermenstrual spotting is reported in up to 25% of patients using the LNG-IUD [8]. Following placement of an IUD it is recommended that its position be confirmed via its threads. Threads may break off or retract into the cervical canal or uterus and missing or shortened threads warrant investigation. It is reasonable to probe the cervical canal using a cytobrush or IUD hook to locate retracted threads, if the threads are not located; the next step is pelvic ultrasound or abdominal X-ray [9].

Multiple imaging and endoscopic modalities were used to try and locate the missing IUD. Thread location, bleeding pattern, and effective contraception suggested the IUD was within the uterus. Ultrasound suggested there was no perforation and provided a specific assessment of the distance from IUD arm to the serosal surface. Hysteroscopy and cystoscopy were not helpful in localizing the IUD. MRI suggested an intrauterine location with perforation of only a single arm of the IUD. Ultimately, laparoscopy was successful in localization and removal.

Modern IUDs are safe and effective. Perforation is an uncommon but serious complication that should be considered whenever threads are not visible at the cervix. While partial or total perforation is most likely to occur during IUD insertion, migration of a normally placed IUD is certainly possible. Uterine contractions are a likely mechanism for migration which in part explains the higher expulsion rate for IUDs placed during the postpartum period [10]. In addition, a normally placed IUD would most likely be expelled through the cervix unless a path of lesser resistance existed. There is no way to confirm when or why perforation occurred in this case. The clinician was experienced, the uterus was mobile and axial in position, and no myometrial defects were noted on imaging or endoscopy. The only known risk factor for perforation was placement during lactation.

Removal was complicated by adhesions and fibrosis suggesting that perforation had occurred at least one week prior to removal. Adhesion formation typically begins within days of tissue injury and becomes dense and organized within one week [11]. While the IUD must have at least partially perforated the uterine wall at some point between insertion and complete perforation, it is unlikely that it was dislodged during hysteroscopy as a clear view of the endocervical canal, lower uterine segment, and endometrial cavity was maintained during the procedure.

The majority opinion within our department suggested continued attempts at removal via thread traction. Our laparoscopic findings should serve as a caution to this approach. The site of perforation was about 1 cm from the right uterine artery. While the pull-through force necessary to divide myometrium is unknown, the proximity of a missing IUD and its retrieval threads to a major vessel is a concern.
